# Acute Neuromuscular Activity in Selected Injury Prevention Exercises with App-Based versus Personal On-Site Instruction: A Randomized Cross-Sectional Study

**DOI:** 10.1155/2019/1415305

**Published:** 2019-10-08

**Authors:** M. K. Zebis, C. Sanderhoff, L. L. Andersen, L. Fernandes, M. Møller, E. Ageberg, G. Myklebust, P. Aagaard, J. Bencke

**Affiliations:** ^1^Department of Physiotherapy, Faculty of Health and Technology, University College Copenhagen, Copenhagen, Denmark; ^2^Human Movement Analysis Laboratory, Copenhagen University Hospital, Amager-Hvidovre, Copenhagen, Denmark; ^3^National Research Centre for the Working Environment, Copenhagen, Denmark; ^4^Sport Sciences, Department of Health Science and Technology, Aalborg University, Aalborg, Denmark; ^5^Department of Sports Science and Clinical Biomechanics, University of Southern Denmark, Odense, Denmark; ^6^Department of Health Sciences, Lund University, Lund, Sweden; ^7^Oslo Sport Trauma Research Center, Norwegian School of Sport Sciences, Oslo, Norway; ^8^Institute of Sports Science and Clinical Biomechanics, Muscle Physiology and Biomechanics Research Unit, University of Southern Denmark, Odense, Denmark

## Abstract

**Introduction:**

A significant step towards sport-related injury prevention is the introduction of easily accessible smartphone applications (apps). However, it is unknown whether this type of app-based instruction facilitates similar acute neuromuscular and biomechanical characteristics of the preventive exercises as achieved when instructed on-site by an expert. Thus, the aim was to evaluate acute neuromuscular characteristics observed during a single bout of selected lower extremity injury preventive exercises instructed by an on-screen app versus on-site individual instruction provided by a physiotherapist.

**Methods:**

In a cross-sectional study design, 47 female football and handball players were randomly assigned to receive app instruction (APP group) or on-site instruction provided by a physiotherapist (PHY group) while performing five lower extremity injury preventive exercises. The exercises performed comprised (1) one-legged balance on Airex, (2) vertical drop jump, (3) one-legged horizontal jump onto floor, (4) one-legged horizontal jump onto Airex, and (5) two-hand Kettlebell Swing. Primary outcome was hamstring (biceps femoris and semitendinosus) muscle activity. Secondary outcomes were quadriceps (vastus lateralis and medialis) muscle activity, as well as hip and knee joint angles. Muscle activity was monitored by surface electromyography (EMG) and normalized to the peak amplitude obtained during a maximal voluntary isometric contraction (MVC). Hip and knee joint angles were recorded by a 3D motion analysis system. A linear mixed model was used to evaluate the differences between experimental conditions for each outcome variable.

**Results:**

Medial hamstring (semitendinosus) muscle activity was significantly higher during one-legged jump onto Airex (17 percentage points (95% CI 7 to 27)) and Kettlebell Swing (19 percentage points (95% CI 2 to 36)) in the PHY group than the APP group. Likewise, the PHY group demonstrated 18 percentage points (95% CI 1 to 35) and 19 percentage points (95% CI 0 to 38), greater lateral quadriceps muscle (vastus lateralis) activity during one-legged jump onto floor and one-legged jump onto Airex, respectively, compared with that of the APP group.

**Conclusions:**

Complex exercises, i.e., Kettlebell Swing and one-legged jump onto Airex, are characterized by lower neuromuscular activity when using app-based instructions compared with on-site instruction provided by a physiotherapist. However, the effectiveness of app-based instruction versus on-site individual instruction in injury prevention interventions remains to be investigated in future longitudinally studies.

## 1. Introduction

Female athletes participating in team sports like football and handball are at high risk of sustaining lower extremity injuries [1, 2]. Strong evidence exists that neuromuscular training can reduce this injury risk among female athletes [3, 4]. The main focus of effective lower-extremity injury prevention programs is to improve control of hip, knees, and ankles during standing, running, cutting, jumping, and landing [5, 6]. Neuromuscular alterations in established and consistent motor programs during sports specific tasks have been associated with preventive exercises that induce high levels of neuromuscular activity in the muscles protecting the joints, e.g., hamstring muscles [7–9].

Despite strong evidence for the efficacy of neuromuscular training, lack of adherence, adoption, and sustainability of such programs are of major concerns [3, 10]. Thus, the overall incidence of lower-extremity injury in female sports has not changed markedly during the last decades [11, 12] despite the introduction of effective preventive programs [5, 6].

Factors associated with successful implementation of injury preventive training include the presence of active support staff [10, 13]. Thus, the ideal intervention is provided via instructions and supervision by a skilled physiotherapist, exercise physiologist, or similar type of trained health professional. However, within the economic constraints of a real-world amateur setting, the majority of athletes do not have access to designated support staff. Thus, alternative and cost-effective options are needed.

E-health and self-management are suggested to represent a cost-effective way to reach and guide the sports active population in the prevention of sports-related injuries. One significant step towards self-managed prevention of sports-related injuries is the introduction of easily accessible smartphone applications (apps) [6, 14]. One example is “Get Set—Train Smarter,” which was released by the Oslo Sports Trauma Research Center and the Norwegian School of Sports Sciences for the occasion of the 2014 Youth Olympic Games in Nanjing, China [14]. The app aims to prevent musculoskeletal injuries in numerous sports by providing the most effective and evidence-based workout routines. All exercises in this app are presented in videos supported by short-written descriptions (in several languages) on how to perform each exercise correctly [14]. The Get Set app is accessible for free and covers 45 different sports, and the exercises are designed to be carried out with a minimum of equipment, to make them safe and easy to implement. Thus, the Get Set app has the potential to reach out to a large population of athletes.

However, it is unknown whether this type of app-based instruction facilitates similar neuromuscular and biomechanical characteristics of the injury preventive exercises as achieved when the athlete is instructed and supervised by an expert instructor (e.g., physiotherapist).

Therefore, the aim of the present study was to evaluate the neuromuscular activity of hamstrings and quadriceps muscles as well as the biomechanical characteristics observed during an acute bout of selected injury preventive exercises when instructed by an app (in accordance with “Get Set—Train Smarter”) versus on-site individual instruction by a physiotherapist.

## 2. Methods

### 2.1. Study Design and Participants

**Figure 1:**
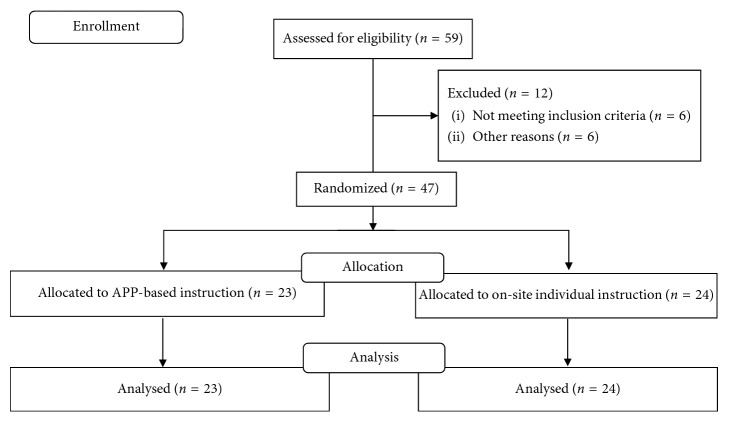
Flowchart describing the inclusion and flow of participants throughout the study.

This study was a cross-sectional study, recording and analyzing neuromuscular activity and biomechanical characteristics during five injury prevention exercises. The subjects were randomly allocated either to receive instructions and supervision on the exercises from a trained physiotherapist (PHY group) or from an app (APP group) (Figure 1). Tests, exercises, and instructions were carried out within one day. The study was performed at the Human Movement Analysis Laboratory, Copenhagen University Hospital, Denmark between September 2015 and January 2016.

Female players from 25 female handball and football teams, located in the Copenhagen area, Denmark, were invited to participate in the study. In total, 59 players, representing 12 different teams, agreed to participate (Figure 1). In a phone interview, players were asked about training habits, injuries, and their knowledge of injury prevention and the five exercises to be examined. Inclusion criteria were ≥18 years old and competing at subelite level (i.e., 2-3 weekly training sessions). Exclusion criteria were previous traumatic knee injury, lower limb injury at the time of testing, and/or previous participation in regular injury prevention training.

All participants were informed about the purpose and content of the project and provided their written informed consent to participate in the study in accordance with the Declaration of Helsinki. The Committees on Biomedical Research Ethics for the Capital Region of Denmark did not consider the study as a health research study, and therefore the study did not need to be notified for full ethical evaluation by the committee (Journal number 15007560). The study was registered in ClinicalTrials.gov (NCT03063814).

### 2.2. Test Protocol and Procedures

All participants were tested in a clinical 3D motion analysis laboratory at a single occasion. On the test day, the participant was first introduced to the laboratory and the test protocol.

The test protocol had a duration of 2 hours and consisted of six procedures (in chronological order): (1) measurement of anthropometric data (age, height, weight, and determining dominant leg), (2) positioning of bipolar EMG-electrodes, (3) standardized warm-up procedure, (4) test of maximum voluntary muscle contraction (MVC), (5) positioning of reflective markers, and (6) test of exercises in a random order sequence.

### 2.3. Five Selected Injury Prevention Exercises

All five exercises were performed by both groups. Participants were allowed to perform as many trials as deemed necessary by the physiotherapist (PHY group) or the participant (APP group) up to a maximum of 10 trials. No participant exceeded 10 trials before having three trials approved.

In PHY group, the same physiotherapist demonstrated and explained the focus areas of each of the five exercises before the participants performed the exercises. If needed, verbal feedback was given between trials to correct the performance of the exercise. The physiotherapist approved trials that were performed with proper technique.

In the APP group, a video sequence of each exercise showing correct exercise performance, supported by short written descriptions, was presented to the participants on an iPad. The person illustrating (video-recorded) the exercises in the app was identical to the physiotherapist who supervised all participants in the PHY group. If required, the participants in the APP group were allowed to watch the video and description several times between trials of the same exercise. The participants approved their own trials when they believed that the exercises had been performed as described in the app.

**Figure 2:**
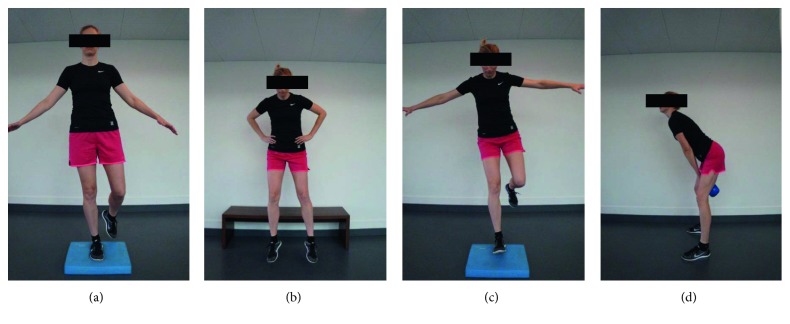
Illustrations of four of the five examined exercises: (a) one-legged balance on Airex, (b) vertical drop jump, (c) one-legged jump on Airex, and (d) Kettlebell Swing. One-legged jump on the floor is not illustrated but resembles one-legged balance on Airex without the Airex mat.

**Figure 3:**
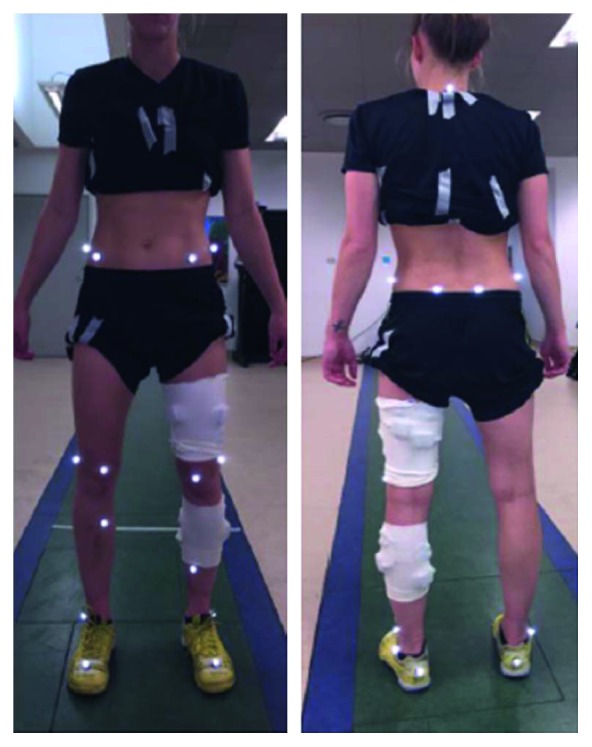
Illustration of joint and segment marker positions.

Five different exercises were examined: (1) one-legged balance on an Airex mat (Figure 2(a)), (2) vertical drop jumping (Figure 2(b)), (3) one-legged forward jump landing on the floor, and (4) one-legged forward jump landing on an Airex mat (Figure 2(c)). The principles of these four balance/coordination and jump exercises are included in the “Get Set—Train Smarter” app [14], and the exercises have previously been included in effective lower extremity injury prevention [5, 15] and rehabilitation programs [16]. In addition, the Kettlebell Swing (Figure 2(d)) was included as the fifth exercise in the present protocol as it is a highly potent exercise to markedly activate the medial hamstring muscle (M. semitendinosus) as supported by the observation of very high semitendinosus EMG activity during this exercise [17]. Since the semitendinosus muscle represents an important functional agonist/synergist to the anterior cruciate ligament (ACL) [8], the Kettlebell Swing appears to be a relevant exercise to be included in future lower extremity injury prevention programs.

The five exercises were performed as follows:

  One-legged balance on Airex: the subjects were instructed to perform single-leg static standing on the preferred push-off leg on a balance mat (40 × 50 cm; 7 cm thick; Alusuisse Airex, Sins, Switzerland, 2000). The subject focuses on knee joint control, i.e., keeping the knee in line over the foot (second toe) and maintains postural balance for 10 seconds. The app instruction lines were as follows: (1) slight bend in knee and hip, (2) keep knee over second toe, and (3) maintain balance for 10 seconds.
  Vertical drop jump: the subjects performed bilateral “drop jumps” by dropping from a 35.5 cm high box to land onto two force plates (one for each leg) which immediately was followed by a maximal vertical jump take-off, followed by a flight phase and a second landing phase. In the landing phases, the subject focuses on stabilizing the knee—the knee over second toe position—and in the final landing to maintain postural balance for 5 seconds. App instruction lines were as follows: (1) step down from the box and jump as high as you can, (2) perform a soft second landing, and (3) ensure that hip-knee-toe in line.
  One-legged horizontal jump onto the floor: the subjects were instructed to perform a horizontal jump with a single-leg landing onto a force plate (individualized to correspond to a distance of 80% leg length) with focus on stabilizing the knee in the sagittal and frontal planes—ensuring alignment of the knee over second toe position—in the landing and maintaining balance for 5 seconds. The app instruction lines were as follows: (1) one-leg jump onto force plate, (2) perform soft landings by bending hips and knees, (3) keep knee positioned over second toe, and (4) maintain balance for 5 seconds.
  One-legged horizontal jump onto Airex: the subjects were instructed to perform a horizontal jump onto a balance mat placed on a force plate (distance of 80% leg length) with focus on stabilizing the knee in the sagittal and frontal planes—the knee over second toe position—in the landing and maintaining balance for 5 seconds. The corresponding app instruction lines were as follows: (1) perform one-leg jump onto balance mat, (2) soft landing bending hips and knees, (3) keep knee over second toe, and (4) maintain balance for 5 seconds.
  Two-hand Kettlebell Swing: this exercise was performed using a 12 kg Kettlebell. The subject was positioned in front of the Kettlebell with feet parallel a shoulder width apart. By flexing the hips and knees while keeping the spine in a neutral position, the subjects were instructed to reach down and grasp the Kettlebell using both hands. The subject forcefully swung the Kettlebell back between the legs and quickly reverses the direction with an explosive extension of the hips swinging the Kettlebell out to chest level where the hips and knees are extended and the subject is standing upright. App instruction text was as follows: (1) keep feet shoulder width apart, (2) keep your back straight and knees nearly straight, (3) swing the Kettlebell forcefully back between your legs by bending in the hips, and (4) reverse the direction by explosively straitening in the hips.

For the exercises 1–4, three approved trials were collected for muscle EMG activity and joint kinematics, and an average of the three trials, respectively, was used for data analysis. For Kettlebell Swing, one approved trial involving 10 successive swings was recorded. The first two, and the last three swings, were excluded, and the middle five swings were averaged and used for analysis.

### 2.4. Outcome Variables

The primary outcome variable was normalized (to isometric MVC peak amplitude) hamstring muscle EMG amplitude (semitendinosus and biceps femoris) recorded during the various five exercises. Secondary outcome variables were EMG amplitude of the quadriceps muscle (vastus lateralis and vastus medialis normalized to isometric MVC peak amplitude) as well as kinematic variables (hip and knee joint angles) recorded in the same exercises.

### 2.5. EMG and 3D Analysis

All exercises were recorded (500 Hz) in a 3D motion analysis laboratory using 8 infrared Vicon T40 cameras (Vicon Motion Systems Ltd, Oxford, UK) and 2 force platforms (model OR 6-7, AMTI, Boston MA, USA). All joint angles were obtained in 3D using a modified Helen-Hayes marker model (Figure 3) and processed by the Plug-in Gait algorithm provided in the motion analysis software (Vicon).

EMG recordings were obtained synchronously with 3D joint kinematic data and vertical ground reaction force (AMTI force plate, Massachusetts, USA). EMG signals from four muscles in the preferred push-off leg (M. semitendinosus: ST; M. biceps femoris: BF; M. vastus medialis: VM; M. vastus lateralis: VL) were analog/digital sampled (1000 Hz) using wireless bipolar electrodes (Myon, Prophysics, Zürich, Switzerland). Prior to electrode placement, the skin of the subject was shaved with a hand razor and carefully cleaned with ethanol. Bipolar surface EMG electrodes (Neuroline 720 01-K, Medicotest A/S, Olstykke, Denmark) were placed according to standardized procedures [18].

All raw EMG signals were prepared for later offline analysis by highpass filtering with a cutoff frequency of 20 Hz and subsequent lowpass filtering using a moving (1-ms steps) root-mean-square filter with a 30 ms time constant, using custom-made algorithms written in Matlab (MathWorks Inc, Natick, MA, USA). After a standardized warm-up program and prior to the exercise test protocol, maximal muscle activation levels (MVC EMG amplitudes) were obtained by performing 3 trials of maximal isometric contractions (MVC) for each muscle group (more details given below). The peak EMG amplitude value from each muscle was used for normalization of muscle activation levels obtained during the respective exercise tests—termed nEMG (i.e., normalized EMG).

In one-legged balance on Airex, the mean nEMG activity during the 5-second sampling of each muscle was used to describe the overall activity level of the exercise.

In drop jump, one-legged horizontal jump onto the floor, and one-legged horizontal jump onto Airex, the mean nEMG activity for each muscle was obtained during the last 50 ms before initial foot contact (IC). For all three jumping exercises, sagittal knee and hip joint angles were recorded.

In Kettlebell Swing, the peak nEMG activity within the entire swing phase was identified and used for further analysis. In addition, maximal knee and hip joint flexion angles were identified within the swing phase.

### 2.6. Maximum Voluntary Isometric Contraction (MVC)

Each participant was instructed and monitored by the same tester (not blinded to group allocation). Before measuring the maximal voluntary isometric contraction (MVC), all participants went through a standardized warm-up procedure consisting of ten countermovement jumps (individualized to correspond to 50% of max effort), ten one-leg squats on each leg, ten countermovement jumps (80% of max effort), ten lunges on each leg, and finally ten maximal countermovement jumps (100% effort).

Knee extensor MVC EMG activity was obtained with the participant sitting on the edge of an examination bench with 90° of hip flexion and 60° of knee flexion (0° = full knee extension), a strap (attached to the bench) wrapped around the ankle, and then performing a maximal isometric knee extensor contraction. The hands were holding on to each side of the bench during testing.

Knee flexor MVC EMG activity was obtained with the participant positioned prone on the examination bench at 10° knee flexion, the ankle free of the bench's edge, a strap (attached to the floor) wrapped around the ankle, and then performing a maximal isometric knee flexor contraction. The hands were holding on to each side of the bench during testing.

All MVC test contractions lasted 4 seconds to allow for maximal muscle activation, and strong verbal encouragement was given to the subjects. Three MVC trials were performed for each muscle with a 30-second rest between each trial to avoid fatigue accumulation.

### 2.7. Randomization and Blinding

The participants were randomized to either the PHY group or the APP group stratified for sport (i.e., handball and football). Thus, two concealed opaque envelopes were made beforehand—one for each sport—from which the participant drew the group allocation. A third envelope was prepared in advance with the random order sequence of the exercises. The randomization protocol was carried out by use of http://www.random.org. To avoid fatigue during testing, the Kettlebell Swing exercise was located as the final exercise in the protocol. The present study design did not allow for blinding of participants or test leader. However, the researcher performing the statistical analyses was blinded to group allocation and sequence of exercises.

### 2.8. Sample Size Calculation

For the primary outcome (hamstring muscle EMG activity), an a priori sample size calculation showed that a minimum of 17 participants in each group would be required to detect a clinically relevant between-group difference of 15% points of nEMG amplitude [7], for a SD of 15%, *α* level of 5%, and statistical power (*β*) of 80%.

### 2.9. Statistical Analysis

A linear mixed model (Proc Mixed, SAS version 9, SAS Institute, Cary, NC) was used to evaluate the differences between experimental conditions for each outcome variable examined. Fixed factors were exercise (one-legged balance on Airex, drop jump, one-legged jump onto the floor, and one-legged jump onto Airex), instruction (PHY and APP), and exercise by instruction interaction. A similar analysis was performed for the KS, as this exercise was not part of the randomized order of exercises and considered an add-on to the four exercises included in previous evidence-based prevention programs. The analyses were controlled for age (continuous), BMI (continuous), pelvic width (continuous), leg length (continuous), and sports (handball and football). The subject was entered in the model as a random factor. Values are reported as least square means (95% confidence interval) unless otherwise stated. *P* values ≤0.05 were considered statistically significant (two-tailed).

## 3. Results

### 3.1. Study Flow

Of 59 eligible female players, six players were excluded due to not fulfilling the inclusion criteria, four players were injured during match play prior to the testing day, and two players did not appear on the day of testing. In total, 47 female players (27 handball and 20 football players) were included in the study and completed the test protocol (Figure 1). None of the included players reported any pain or discomfort prior to, during, or after testing. Anthropometrics and sports participation characteristics are presented in Table 1.

### 3.2. Main Effect (Group × Exercise)

Significant *group* × *exercise* interactions were observed for M. semitendinosus nEMG activity (*P*=0.04), M. vastus lateralis nEMG activity (*P*=0.03), and knee flexion angle at IC (*P*=0.05). Outcome measures for each exercise are presented in Table 2. For significant *group* × *exercise* interactions, post hoc tests are presented in the following.

### 3.3. Semitendinosus Muscle Activity

**Figure 4:**
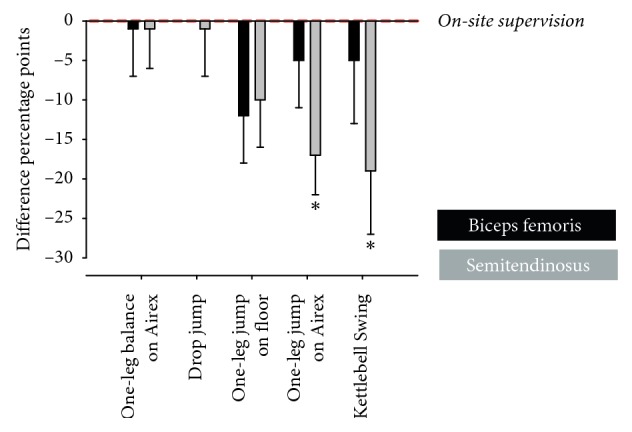
Differences in hamstring muscle activation between app-based instruction and on-site individual instruction for the five selected injury prevention exercises. Data are presented relative to on-site individual instruction (red dotted line). Black bars represent semitendinosus nEMG activity with app-based instruction. Gray bars represent biceps femoris nEMG activity with app-based instruction. ^*∗*^App-based instruction vs. on-site instruction (*P* ≤ 0.05).

The nEMG activity of M. semitendinosus during the one-legged horizontal jump onto Airex was significantly higher in the PHY group than the APP group (17 percentage points (95% CI 7 to 27); *P*=0.002) (Table 2). Likewise, a tendency towards elevated M. semitendinosus nEMG activity during one-legged horizontal jump onto floor was observed in the PHY group compared with the APP group (10 percentage points (95% CI −1 to 22); *P*=0.076) (Table 2). During Kettlebell Swing, 19 percentage points higher m. semitendinosus nEMG activity was observed in the PHY group than the APP group (95% CI 2 to 36, *P*=0.03) (Table 2, Figure 4). No differences between PHY and APP groups were observed during execution of one-legged balance on Airex or drop jump tasks (Table 2).

### 3.4. Vastus Lateralis Muscle Activity

During one-legged horizontal jump onto floor and one-legged horizontal jump onto Airex, 19 percentage points (95% CI 0 to 38; *P*=0.05) and 18 percentage points (95% CI 1 to 35; *P*=0.04) higher nEMG muscle activity, respectively, were observed in the PHY group compared with the APP group. No PHY vs APP differences in nEMG M. vastus lateralis activity were observed for any of the remaining exercises.

### 3.5. Knee Joint Kinematics

During one-legged horizontal jump onto Airex testing, a 4-degree more flexed knee joint angle was observed at initial foot contact in the PHY group compared with the APP group (15° (95% CI 12 to 17) vs. 11° (95% CI 9 to 14); *P*=0.05). During Kettlebell Swing, the PHY group demonstrated a more extended knee joint angle during the swing phase compared with the APP group (42° [95% CI: 37 to 48] vs. 55° [95% CI: 49 to 61]) (*P*=0.003) (Table 2).

## 4. Discussion

The present study demonstrates that, in female athletes performing complex knee injury preventive exercises (i.e., one-legged horizontal jump onto Airex and Kettlebell Swing), a pattern of increased medial hamstring and lateral quadriceps muscle activity was observed when instructions were provided by an on-site skilled person (i.e., physiotherapist) compared to app-based instructions (Figure 4). This observation is important as the medial hamstring is considered an essential dynamic knee joint stabilizer [9] and inadequate capacity to activate the medial hamstring muscle during an explosive sidecutting movement has been shown to represent a potential risk factor for one of the most serious lower extremity injuries, i.e., noncontact ACL injury [8].

In a one-legged landing situation, the importance of adequately adjusted quadriceps-hamstring muscle activity is to avoid the “straight leg position,” which is known to increase the anterior shear force of the tibia [19], thus increasing the magnitude of ACL loading. In the present study, individual on-site instruction (PHY group) led participants to adopt a more flexed knee joint position at the instant of foot contact compared with app-based instruction (APP group). Thus, the more flexed knee joint position in combination with enhanced semitendinosus and vastus lateralis activity may have enabled athletes in the PHY group to land in a position less likely to load the ACL.

Although one-legged landing on a stable surface (i.e., floor) is less complex than landing on an unstable surface (i.e., Airex), the one-legged horizontal jump onto floor exercise displayed the same differences in muscle activity between the two instruction modes as observed for the one-legged horizontal jump onto Airex exercise. Interestingly, however, when the surface was stable (less demanding), no significant difference between groups was observed in knee flexion at initial landing.

In the Kettlebell Swing exercise, individual on-site instruction by a physiotherapist (PHY group) resulted in a more correct performance with reduced magnitude of knee flexion compared with app-based instruction (Table 2). Smaller knee joint flexion angles (i.e., more extended knee joint positions) facilitate more stretched hamstring muscles where the load is highest, i.e., at the point of reversal from eccentric to concentric hamstring muscle actions. The medial hamstring, M. semitendinosus, is, in contrast to the lateral biceps femoris, parallel fibered with long fiber lengths and a high number of sarcomeres in series [20]. This arrangement increases the total shortening capacity (ROM) and absolute velocity of contraction of the semitendinosus muscle [21] and potentially provides this muscle with superior working conditions at long muscle lengths compared to the biceps femoris muscle. The present findings suggest that to secure proper exercise technique (and hence ensure optimal exercise stimulation) in novice athletes, the Kettlebell Swing exercise should be thoroughly instructed to the athlete prior to self-management. Although instruction from a physiotherapist appears to elicit reduced knee flexion in the Kettlebell Swing exercise than seen with app-based instruction, the PHY group displayed markedly more knee flexion (on average 42°) than previously observed among female elite athletes (on average 7°) during execution of Kettlebell Swing [17]. Although the efficacy of the Kettlebell Swing as an injury preventive exercise remains to be validated, its eligibility is supported by the involvement of the semitendinosus muscle to prevent noncontact ACL injury [8].

The least complex exercise examined, i.e., one-legged balance on Airex, displayed no difference in muscle activity between instruction modalities, indicating that this exercise is simple to implement in app-based injury prevention programs. However, in line with previous study observations [22], the one-legged balance on Airex exercise was characterized by low muscle activity levels in the hamstring muscles (as well as in the quadriceps muscle), and it is therefore not deemed likely that this exercise would induce increased hamstring activity levels during typical sports-related injury risk situations. Thus, the one-legged balance on Airex exercise may not serve a distinct injury preventive purpose per se, unless the exercise is used as a rehabilitation exercise in the early stage after ACL-reconstruction where limited ROM and muscle activity typically is tolerated [23] or the training focus lies within prevention of ankle joint injuries [24].

The drop jump exercise has been implemented in validated lower extremity injury prevention programs [5, 15], with focus on proper landing technique without excessive frontal plane knee joint movements, i.e., dynamic knee joint valgus. The present findings that normalized EMG activity and knee joint kinematics were similar in this exercise between on-site instruction from a physiotherapist and app-based instruction modalities may imply that the drop jump exercise is well suited for future app-based injury prevention programs.

The present study findings indicate that to optimally activate the thigh muscles, especially the medial hamstring, during the execution of complex exercises, stand-alone app-based instruction with fixed single-plane video shots appears insufficient. Thus, instruction videos recorded in both the sagittal and frontal planes during the execution of complex exercises—in addition to a bullet line suggesting, e.g., a mirror as feedback—may increase the efficacy of the exercises when performed by novice athletes. Alternatively, incorporating a single on-site instruction from an expert when starting up app-based exercising may ensure proper muscle activity and kinematics. Another solution may be to develop apps that use wearables connected to smartphones to provide biofeedback of lower limb joint positions during the exercises performed, which enables immediate movement corrections.

The implementation of musculoskeletal injury prevention by use of app-based instruction videos is a promising way to increase adherence among athletes as it is easily accessible, and the implementation cost is low. However, the implementation of evidence-based apps has been found to be challenging, and more targeted efforts may be required to ensure proper uptake and usage of the app by the target population [25]. In addition, in the achievement of optimal app-based instructions the present study underlines the importance of validating app-based instructions to ensure sufficient efficacy of the exercises performed.

The present study has several potential limitations. One potential limitation was that the inherent biological and methodological variance in surface EMG recording makes it difficult to evaluate subtle differences in muscle activity. Further, it would have strengthened the present data if the participants had rated their perceived difficulty in performing the different exercises to support the findings on exercise complexity. We cannot exclude that other exercises would have provided different results. However, the evaluation covers both simple as well as complex exercises, which may increase the external validity. Finally, as the present study evaluated the exercises among female subelite athletes >18 yrs old with no previous participation in regular injury prevention training, the findings may not be readily transferrable to other sports populations or younger age groups.

## 5. Conclusion

The present study indicates that simple exercises like one-legged balance on Airex and drop jump induce the same neuromuscular stimuli regardless of instruction type. In contrast, complex exercises like the Kettlebell Swing and one-legged jump on Airex are characterized by lower muscle activity in the important ACL-synergist, M. semitendinosus, when implemented by app-based instructions compared with on-site individual instructions. However, the effectiveness of app-based instruction versus on-site individual instruction in injury prevention interventions remains to be investigated in future longitudinally studies.

## Figures and Tables

**Table 1:** Participant characteristics.

	APP (*n* = 23)	PHY (*n* = 24)	*P* value
Age (years)	23.2 (2.9)	23.1 (4.6)	0.332
Weight (kg)	68.5 (9.1)	68.1 (7.8)	0.888
Height (cm)	172 (1)	170 (1)	0.476
Sport experience (years)	13.0 (3.6)	14.3 (4.1)	0.274
Training exposure (hrs/wk)	5.0 (1.1)	4.4 (1.1)	0.096
Strength training (hrs/wk)	1.2 (1.4)	1.0 (1.3)	0.598
Handball : football (*n*)	13 : 10	14 : 10	

APP: group receiving app-based instruction. PHY: group receiving instruction from a physiotherapist. Values displayed as mean (SD).

**Table 2:** Normalized EMG and kinematics during exercises presented as mean (95% CI) in APP (*n* = 23) and PHY (*n* = 24) group, respectively.

	One-leg balance on Airex		Drop jump		One-leg jump on the floor		One-leg jump on Airex		Kettlebell swing	
	APP	PHY	APP	PHY	APP	PHY	APP	PHY	APP	PHY
Biceps femoris (% EMGmax)	2 (0–11)	3 (0–12)	10 (0–19)	10 (1–19)	21 (11–30)	34 (25–43)	38 (29–46)	43 (34–51)	49 (40–58)	54 (46–62)
Semitendinosus (% EMGmax)	2 (0–9)	3 (0–10)	10 (1–18)	11 (3–18)	20 (11–28)	30 (22–38)^§^	32 (24–39)	49 (41–56)^*∗*^	41 (28–53)	60 (48–71)^*∗*^
Vastus lateralis (% EMGmax)	5 (0–17)	7 (0–19)	29 (15–43)	19 (7–31)	32 (18–47)	52 (39–65)^*∗*^	39 (27–52)	57 (45–70)^*∗*^	56 (42–70)	50 (38–62)
Vastus medialis (% EMGmax)	4 (0–13)	7 (0–15)	18 (9–28)	20 (11–28)	33 (24–42)	36 (27–45)	30 (21–38)	40 (32–49)^§^	51 (42–59)	50 (42–58)

Knee flexion angle at IC (°)	—	—	24 (22–27)	22 (20–25)	10 (8–13)	13 (10–15)	11 (9–14)	15 (12–17)^*∗*^	—	—
Knee flexion angle max (°)	—	—	85 (81–90)	90 (87–94)	53 (49–57)	57 (53–61)	51 (48–55)	55 (51–59)	55 (49–61)	42 (37–48)^*∗*^
Knee valgus angle at IC (°)	—	—	5 (4–6)	4 (3–5)	4 (3–5)	2 (1–3)	3 (2–4)	2 (1–3)	—	—
Knee valgus angle max (°)	—	—	2 (1–3)	0 (−1-1)	4 (2–5)	2 (1–3)	3 (2–4)	1 (0–2)	—	—
Hip flexion angle at IC (°)	—	—	31 (27–35)	32 (28–36)	36 (32–40)	41 (37–45)	40 (37–44)	44 (41–48)	—	—
Hip flexion angel max (°)	—	—	81 (75–86)	89 (85–94)	55 (50–60)	58 (53–63)	54 (50–59)	58 (53–62)	80 (74–86)	83 (78–89)
Hip internal rotation at IC (°)	—	—	−3 (−6–0)	−6 (−9–(−3))	−1 (−4–3)	−2 (−5–2)	2 (−1–5)	3 (−1–6)	—	—
Hip internal rotation max (°)	—	—	12 (9–15)	11 (8–14)	4 (1–7)	3 (0–6)	5 (2–8)	4 (2–7)	—	—

APP: group receiving app-based instruction. PHY: group receiving instruction from a physiotherapist. CI: confidence interval. IC: initial contact. ^*∗*^Significance level at *P* ≤ 0.05. ^§^Significance level at *P* < 0.1.

## Data Availability

The data used to support the findings of this study are available from the corresponding author upon request.
